# Aging with leukemia: public health strategies for optimizing quality of life and palliative care in refractory cases

**DOI:** 10.1097/MS9.0000000000003815

**Published:** 2025-09-01

**Authors:** Emmanuel Ifeanyi Obeagu, Aakib Rahman Parray

**Affiliations:** a Department of Biomedical and Laboratory Science, Africa University, Mutare, Zimbabwe; b Department of Psychology, Akal University, Punjab, India

**Keywords:** aging, leukemia, palliative care, public health strategies, quality of life

## Abstract

Leukemia incidence increases with age, and managing refractory cases in older adults poses unique clinical and public health challenges. Aging patients with leukemia often experience a complex symptom burden exacerbated by comorbidities, frailty, and psychosocial factors that diminish their quality of life (QoL). Traditional curative treatments may become less effective or inappropriate, shifting the focus toward comprehensive supportive care and palliative interventions to alleviate symptoms and enhance well-being. This review examines public health strategies aimed at optimizing QoL and palliative care delivery for older adults with refractory leukemia. Emphasis is placed on multidisciplinary care models that integrate hematology, geriatrics, palliative services, and social support. Addressing social determinants of health, expanding community-based programs, and leveraging telemedicine are highlighted as crucial elements to improve care access, reduce hospitalizations, and support aging patients in their preferred settings. Furthermore, the review identifies key barriers to effective palliative care, including healthcare system fragmentation, provider knowledge gaps, and stigma surrounding palliative services. Policy recommendations focus on workforce education, early palliative care integration, and equitable resource allocation to better meet the needs of this vulnerable population. Ultimately, coordinated public health efforts are essential to enhance the QoL and dignity for aging individuals living with refractory leukemia.

## Introduction

Leukemia is a heterogeneous group of hematologic malignancies characterized by abnormal proliferation of white blood cells^[1,2]^. Its incidence significantly increases with age, making leukemia predominantly a disease of older adults[3]. As global life expectancy rises and populations age, the burden of leukemia in the elderly is escalating^[4,5]^. This demographic trend presents complex challenges for healthcare systems, as older patients often experience distinct biological, clinical, and social factors that complicate disease management and impact treatment outcomes^[6,7]^. Older adults with leukemia frequently have multiple comorbidities such as cardiovascular disease, diabetes, and cognitive impairment^[8,9]^, which influence both the progression of leukemia and the patient’s ability to tolerate aggressive therapies^[10,11]^. Moreover, physiological changes associated with aging, including immune senescence and reduced organ reserve, increase vulnerability to treatment toxicities and infections^[12,13]^. In refractory leukemia cases, where the disease is resistant to standard therapies, these factors are magnified, resulting in a higher symptom burden and poorer prognosis[14].

**HIGHLIGHTS**

- Elderly leukemia patients face unique care challenges.
- Palliative strategies improve quality of life.
- Early symptom control enhances patient outcomes.
- Multidisciplinary care ensures holistic support.
- Public health planning bridges care gaps.

In refractory leukemia, curative treatment options are often limited or ineffective, and the focus of care naturally shifts toward optimizing quality of life (QoL)^[15–17]^. This shift requires a comprehensive, multidisciplinary approach that goes beyond traditional cancer treatment to include symptom management, psychosocial support, and preservation of functional independence^[18,19]^. Palliative care, which aims to improve QoL by addressing physical, emotional, and spiritual needs, is particularly critical in this context but remains underutilized in hematologic malignancies compared to solid tumors^[20,21]^. Public health strategies have a pivotal role in improving outcomes for older adults living with refractory leukemia by promoting timely access to palliative care, integrating geriatric principles into oncology, and addressing social determinants of health^[22,23]^. Such strategies can enhance care coordination, reduce unnecessary hospitalizations, and support patients in their preferred care settings^[24–26]^. Furthermore, the development of age-specific guidelines and healthcare policies that prioritize QoL is essential for addressing the growing needs of this vulnerable population.

## Aim

The aim of this review is to explore public health strategies that optimize QoL and enhance palliative care delivery for older adults living with refractory leukemia. Specifically, the review seeks to:

- Examine the unique clinical and psychosocial challenges faced by aging leukemia patients with treatment-resistant disease.

- Highlight multidisciplinary care approaches and supportive interventions tailored to the needs of this population.
- Identify barriers to effective palliative care access and utilization in older adults with refractory leukemia.
- Discuss policy and healthcare system reforms necessary to improve equitable access to quality palliative care.
- Provide recommendations for integrating geriatric oncology principles and public health frameworks to better support aging individuals living with leukemia.

## Aging and refractory leukemia: complex care needs

Leukemia in older adults often presents with complex clinical challenges that arise from the interplay between the biology of the disease and the physiological changes associated with aging^[27,28]^. Aging is accompanied by a decline in organ function, reduced bone marrow reserve, and immune system alterations^[29,30]^, all of which influence both the progression of leukemia and the patient’s ability to tolerate intensive therapies^[10,31]^. In refractory leukemia – where the disease does not respond to standard treatments – these age-related vulnerabilities are further magnified, complicating management and worsening outcomes. Comorbidities are common among elderly leukemia patients and significantly affect treatment decisions and prognosis[32]. Conditions such as cardiovascular disease, diabetes, renal impairment, and cognitive decline not only increase the risk of treatment-related toxicities but also impact overall functional status and survival[33]. The presence of frailty, a multidimensional syndrome characterized by decreased physiological reserves and increased vulnerability to stressors, is particularly prevalent in this group. Frailty correlates with higher rates of treatment complications, hospitalization, and mortality, underscoring the need for individualized care plans that carefully balance efficacy with tolerability^[34,35]^.

In refractory leukemia cases, symptom burden can be profound, with patients experiencing persistent fatigue, pain, infections, bleeding, and psychological distress^[36,37]^. These symptoms often lead to frequent healthcare utilization and reduced QoL[38]. The management of these complex needs requires a holistic approach that includes effective symptom control, psychosocial support, nutritional optimization, and rehabilitation services[19]. Importantly, older adults may prioritize QoL and maintenance of independence over aggressive interventions, making patient-centered care planning and shared decision-making critical components of care^[8,39]^. Furthermore, social determinants such as limited caregiver support, social isolation, and financial constraints can exacerbate the challenges faced by aging leukemia patients[40]. Many older adults encounter barriers to accessing appropriate healthcare services, including transportation difficulties and fragmented care coordination[41]. Addressing these social factors is essential to ensure equitable and effective care delivery. Comprehensive geriatric assessments (CGA) are valuable tools in evaluating the multifaceted needs of older leukemia patients[42]. By assessing physical health, cognition, psychological state, social circumstances, and functional ability, CGA facilitates risk stratification and guides tailored interventions[43]. Incorporating CGA findings into treatment planning helps optimize therapeutic choices, mitigate risks, and enhance supportive care efforts[44].

## Public health strategies for optimizing quality of life

Optimizing QoL for older adults living with refractory leukemia demands coordinated public health strategies that extend beyond clinical care to encompass social, psychological, and environmental factors^[22,45]^. Central to these strategies is the early integration of palliative care, which focuses on symptom management, psychosocial support, and maintaining functional independence[46]. Public health initiatives must promote awareness and education about palliative care among healthcare providers, patients, and families to reduce stigma and encourage timely referrals[47]. Community-based programs and home care services play a critical role in supporting aging leukemia patients by providing accessible, patient-centered care in familiar environments^[48,49]^. These programs can reduce unnecessary hospitalizations and emergency visits, enhance medication adherence, and offer psychosocial support to both patients and caregivers[50]. Public health systems should prioritize the development and funding of such services, especially in rural and underserved areas where healthcare access is limited[51].

Addressing social determinants of health is another essential component of public health strategies aimed at improving QoL^[52–54]^. Factors such as socioeconomic status[55], caregiver availability, transportation, housing, and food security significantly influence health outcomes and care engagement^[56,57]^. Policymakers and public health professionals must implement interventions that mitigate these disparities, including caregiver support programs, patient education initiatives, and policies that ensure affordable, coordinated care[58]. Technology-enabled solutions, such as telemedicine and remote patient monitoring, offer promising avenues to extend palliative and supportive care services to older adults who face mobility or transportation challenges[59]. These innovations enable continuous symptom assessment, timely intervention, and improved communication between patients and healthcare teams. Public health systems should invest in infrastructure and training to expand the use of telehealth while ensuring equitable access across diverse populations^[60,61]^.

Furthermore, workforce development is critical for sustaining quality care delivery. Public health strategies should include training healthcare providers in geriatric oncology and palliative care principles to better meet the unique needs of older leukemia patients[62]. Interprofessional education programs can foster collaboration across disciplines, enhancing care coordination and patient outcomes[63]. Data collection and quality improvement initiatives are vital to monitor the effectiveness of public health interventions[64]. Implementing standardized metrics for QoL, symptom control, and healthcare utilization can inform policy adjustments and resource allocation. Engaging patients and caregivers in feedback mechanisms ensures that services remain responsive to their evolving needs.

## Multidisciplinary care models and collaboration

Effective management of aging patients with refractory leukemia requires a multidisciplinary approach that integrates expertise across medical, nursing, psychosocial, and supportive care disciplines[65]. Multidisciplinary care models foster comprehensive assessments and individualized treatment plans that address the complex clinical, functional, and psychosocial needs of older adults^[66–68]^. Such collaboration ensures that care is patient-centered, holistic, and aligned with the goals and preferences of patients and their families. At the core of multidisciplinary care is the collaboration between hematologists and geriatricians[69]. Hematologists provide disease-specific expertise, guiding leukemia management and treatment decisions, while geriatricians assess frailty, comorbidities, cognitive function, and overall physiological reserve[70]. This partnership enables more precise risk stratification and therapeutic tailoring, optimizing both safety and efficacy in treatment planning. Incorporating comprehensive geriatric assessments (CGA) within routine oncology care helps identify vulnerabilities that may influence treatment tolerance and outcomes[71].

Palliative care specialists are essential members of the care team[72], particularly for patients with refractory disease where curative options are limited. Early integration of palliative care supports symptom management, psychosocial support, advance care planning, and end-of-life care discussions[46]. Their involvement helps mitigate physical and emotional distress, facilitating a smoother transition to comfort-focused care when appropriate. Social workers and psychologists contribute by addressing mental health concerns, caregiver burden, and social determinants of health that impact treatment adherence and QoL^[40,73]^. Nurses, including specialized oncology and palliative care nurses, play a critical role in care coordination, symptom monitoring, patient education, and advocacy^[74–76]^. They often serve as the primary point of contact for patients and families, ensuring continuous communication and timely response to emerging issues[77]. Rehabilitation therapists and nutritionists are also valuable team members, aiding in functional maintenance and nutritional optimization, which are key factors in supporting resilience and improving overall well-being^[78,79]^.

The implementation of multidisciplinary tumor boards or care conferences facilitates regular communication and joint decision-making among team members. These forums promote shared understanding of patient status, alignment of treatment goals, and coordinated interventions, reducing fragmentation of care[80]. Furthermore, involving patients and their families in these discussions enhances shared decision-making, empowering patients and respecting their autonomy. Collaboration extends beyond the clinical setting to include community resources and support services[81]. Partnerships with home health agencies, hospice providers, and patient advocacy groups broaden the scope of care and ensure continuity as patients transition between care settings[82]. Telehealth platforms and electronic health records further enhance multidisciplinary communication and information sharing, improving responsiveness and care quality.

## Barriers to palliative care and quality of life improvement

Despite the recognized benefits of palliative care in managing refractory leukemia among older adults, multiple barriers hinder its effective delivery and utilization[83]. These barriers exist at patient, provider, systemic, and societal levels, collectively impacting timely access to comprehensive supportive care and undermining efforts to optimize quality of life. At the patient level, lack of awareness and misconceptions about palliative care often delay acceptance and referral[84]. Many older adults and their families associate palliative care exclusively with end-of-life or hospice services, equating it with giving up on treatment. This stigma and fear can result in reluctance to engage with palliative care teams, leading to unmanaged symptoms, psychological distress, and diminished QoL^[85,86]^. Additionally, cognitive impairments or communication difficulties common in aging populations can complicate discussions about goals of care and advance care planning.

Healthcare providers also face challenges in integrating palliative care for older leukemia patients[87]. Limited training in geriatric oncology and palliative medicine leaves many clinicians uncertain about when and how to initiate palliative interventions. Oncologists may focus predominantly on disease-directed therapies and delay palliative care involvement until late-stage disease, missing opportunities for early symptom management and holistic support^[88,89]^. Moreover, time constraints and fragmented care systems can impede multidisciplinary collaboration essential for comprehensive palliative care delivery. At the health system level, resource limitations and structural barriers further restrict access. Insufficient availability of specialized palliative care teams, especially in rural or underserved regions, limits patient options. Financial constraints and insurance coverage disparities may also hinder the utilization of home-based or community palliative services[90]. Additionally, fragmented care coordination between oncology, primary care, and palliative teams can lead to inconsistent symptom monitoring and gaps in psychosocial support[91].

Societal factors, including cultural beliefs and socioeconomic disparities, influence perceptions of palliative care and access to quality services. In some communities, discussing death and advanced illness remains taboo, reducing openness to palliative approaches^[92,93]^. Economic hardships disproportionately affect older adults, particularly those with limited social support, further complicating their ability to engage in consistent, high-quality care^[94,95]^. Addressing these barriers requires multifaceted interventions. Public health efforts to raise awareness and educate both patients and providers about the benefits and scope of palliative care are vital[47]. Training programs in geriatric oncology and palliative care should be expanded to equip clinicians with the skills to initiate timely, patient-centered discussions. Health systems must prioritize resource allocation to build capacity for community-based and home palliative care services and improve care coordination through integrated health records and multidisciplinary teams^[96–98]^.

## Policy implications

The increasing prevalence of refractory leukemia among older adults necessitates urgent policy attention to address the complex care needs of this vulnerable population. Effective policies must prioritize the integration of geriatric principles and palliative care into leukemia management, ensuring that healthcare systems are equipped to deliver comprehensive, patient-centered care that enhances QoL. One critical policy focus is the development and implementation of national guidelines that incorporate geriatric assessment and palliative care protocols specific to hematologic malignancies. Such guidelines can standardize best practices, promote early identification of patients who would benefit from supportive care, and facilitate individualized treatment planning that balances disease control with QoL considerations. Healthcare financing policies also play a pivotal role. Sustainable funding models are essential to support the expansion of multidisciplinary teams, community-based palliative services, and home care programs. Policies should incentivize healthcare providers to adopt integrated care models through reimbursement structures that recognize the value of palliative interventions and care coordination. Additionally, addressing financial barriers faced by patients, such as copayments and out-of-pocket expenses, is crucial to equitable access.

Workforce development policies must prioritize training in geriatric oncology and palliative care. Investing in continuing education, interdisciplinary training programs, and certification pathways can enhance provider competence and confidence in managing older leukemia patients with refractory disease. Furthermore, policies promoting team-based care and collaborative practice can foster multidisciplinary approaches essential for complex case management. Public health policies should also target social determinants of health that affect aging leukemia patients. By supporting initiatives that improve caregiver resources, transportation, housing stability, and nutritional support, policymakers can help mitigate external factors that compromise treatment adherence and QoL. Data infrastructure and health information policies must encourage the collection of age-specific, disease-relevant metrics on outcomes, service utilization, and patient-reported QoL. Robust data enable continuous quality improvement, resource allocation, and evidence-based policy refinements. Additionally, policies ensuring interoperability of electronic health records facilitate seamless communication among care teams, enhancing care continuity. Patient advocacy and engagement policies are vital to ensure that the voices of older adults living with refractory leukemia and their caregivers inform health system design and policy formulation. Empowering patients through education and shared decision-making frameworks aligns care delivery with their values and preferences.

## Conclusion

Aging with refractory leukemia presents a constellation of complex challenges that extend beyond disease management to encompass physical, psychological, and social dimensions. Optimizing QoL and ensuring dignified care for older adults with treatment-resistant leukemia require comprehensive public health strategies, multidisciplinary collaboration, and robust policy support. Early integration of palliative care, tailored interventions based on geriatric assessments, and community-based support services are essential components of an effective care framework. Addressing barriers to palliative care and QoL improvements demands concerted efforts at multiple levels, including enhancing provider education, expanding access to specialized services, and mitigating social determinants that hinder care engagement. Policy initiatives focused on financing, workforce development, and data-driven quality improvement will be critical to sustaining these efforts and ensuring equitable access for all patients regardless of geographic or socioeconomic status.

## Data Availability

Not applicable as this a narrative review.

## Research registration unique identifying number (UIN)

Not applicable as this a narrative review.

## References

[1] Jurlander J. Hematological malignancies, leukemias and lymphomas. In: Jurlander J, ed. Encyclopedia of Cancer. Berlin, Heidelberg: Springer; 2011:1640–44.

[2] Rodriguez-Abreu D Bordoni A Zucca E. Epidemiology of hematological malignancies. Ann Oncol 2007;18:i3–8. 17311819 10.1093/annonc/mdl443

[3] Hao T Li-Talley M Buck A. An emerging trend of rapid increase of leukemia but not all cancers in the aging population in the United States. Sci Rep 2019;9:12070. 31427635 10.1038/s41598-019-48445-1 PMC6700310

[4] Huang P Zhang J. Global leukemia burden and trends: a comprehensive analysis of temporal and spatial variations from 1990—2021 using GBD (Global Burden of Disease) data. BMC Public Health 2025;25:262. 39838416 10.1186/s12889-025-21428-w PMC11753064

[5] Wang W Zhang D Liang Q. Global burden, risk factor analysis, and prediction study of leukaemia from 1990 to 2030. J Glob Health 2024;14:04150. 39173170 10.7189/jogh.14.04150 PMC11345035

[6] Jane Osareme O Muonde M Maduka CP. Demographic shifts and healthcare: a review of aging populations and systemic challenges. Int J Sci Res Arch 2024;11:383–95.

[7] Roller-Wirnsberger R Thurner B Pucher C. The clinical and therapeutic challenge of treating older patients in clinical practice. Br J Clin Pharmacol 2020;86:1904–11. 31321798 10.1111/bcp.14074 PMC7495268

[8] Storey S Gray TF Bryant AL. Comorbidity, physical function, and quality of life in older adults with acute myeloid leukemia. Curr Geriatr Rep 2017;6:247–54. 29479516 10.1007/s13670-017-0227-8 PMC5820770

[9] Hshieh TT Jung WF Grande LJ. Prevalence of cognitive impairment and association with survival among older patients with hematologic cancers. JAMA Oncol 2018;4:686–93. 29494732 10.1001/jamaoncol.2017.5674 PMC5885202

[10] Burnett A Wetzler M Löwenberg B. Therapeutic advances in acute myeloid leukemia. J Clin Oncol 2011;29:487–94. 21220605 10.1200/JCO.2010.30.1820

[11] Blagosklonny MV. Why therapeutic response may not prolong the life of a cancer patient: selection for oncogenic resistance. Cell Cycle 2005;4:1693–98. 16294046 10.4161/cc.4.12.2259

[12] Nigam Y Knight J Bhattacharya S. Physiological changes associated with aging and immobility. J Aging Res 2012;2012:468469. 22619717 10.1155/2012/468469 PMC3352586

[13] Henry CJ Marusyk A DeGregori J. Aging-associated changes in hematopoiesis and leukemogenesis: what’s the connection?. Aging (Albany NY) 2011;3:643. 21765201 10.18632/aging.100351 PMC3164372

[14] Oliva EN Ronnebaum SM Zaidi O. A systematic literature review of disease burden and clinical efficacy for patients with relapsed or refractory acute myeloid leukemia. Am J Blood Res 2021;11:325. 34540343 PMC8446831

[15] Montserrat E Moreno C Esteve J. How I treat refractory CLL. Blood 2006;107:1276–83. 16204307 10.1182/blood-2005-02-0819

[16] Yan CH Wang Y Sun YQ. Optimized therapeutic strategy for patients with refractory or relapsed acute myeloid leukemia: long-term clinical outcomes and health-related quality of life assessment. Cancer Commun 2022;42:1387–402. 10.1002/cac2.12376 PMC9759766 36274263

[17] Topp MS Zimmerman Z Cannell P. Health-related quality of life in adults with relapsed/refractory acute lymphoblastic leukemia treated with blinatumomab. Blood, J Am Soc Hematol 2018;131:2906–14. 10.1182/blood-2017-09-804658 PMC6024638 29739753

[18] Silver JK Baima J Mayer RS. Impairment-driven cancer rehabilitation: an essential component of quality care and survivorship. CA Cancer J Clin 2013;63:295–317. 23856764 10.3322/caac.21186

[19] Silver JK Raj VS Fu JB. Cancer rehabilitation and palliative care: critical components in the delivery of high-quality oncology services. Supportive Care Cancer 2015;23:3633–43. 10.1007/s00520-015-2916-1 26314705

[20] Gatta B LeBlanc TW. Palliative care in hematologic malignancies: a multidisciplinary approach. Expert Rev Hematol 2020;13:223–31. 32066301 10.1080/17474086.2020.1728248

[21] Meier DE Brawley OW. Palliative care and the quality of life. J Clin Oncol 2011;29:2750. 21670456 10.1200/JCO.2011.35.9729 PMC3139393

[22] Loh KP Abdallah M Kumar AJ. Health-related quality of life and treatment of older adults with acute myeloid leukemia: a young international society of geriatric oncology review paper. Curr Hematol Malig Rep 2019;14:523–35. 31776773 10.1007/s11899-019-00552-6 PMC6938300

[23] Sekeres MA Stone RM Zahrieh D. Decision-making and quality of life in older adults with acute myeloid leukemia or advanced myelodysplastic syndrome. Leukemia 2004;18:809–16. 14762444 10.1038/sj.leu.2403289

[24] Bryan JC Jabbour EJ. Management of relapsed/refractory acute myeloid leukemia in the elderly: current strategies and developments. Drugs Aging 2015;32:623–37. 26286093 10.1007/s40266-015-0285-6

[25] Figueroa JF Feyman Y Zhou X. Hospital-level care coordination strategies associated with better patient experience. BMJ Qual Saf 2018;27:844–51. 10.1136/bmjqs-2017-007597 29618639

[26] Berry LL Rock BL Houskamp BS. Care coordination for patients with complex health profiles in inpatient and outpatient settings. Mayo Clin Proc 2013;88:184–19. 23290738 10.1016/j.mayocp.2012.10.016

[27] Stone RM. The difficult problem of acute myeloid leukemia in the older adult. CA Cancer J Clin 2002;52:363–71. 12469764 10.3322/canjclin.52.6.363

[28] Eleni LD Nicholas ZC Alexandros S. Challenges in treating older patients with acute myeloid leukemia. J Oncol 2010;2010:943823. 20628485 10.1155/2010/943823 PMC2902223

[29] Weyand CM Goronzy JJ. Aging of the immune system. Mechanisms and therapeutic targets. Ann Am Thorac Soc 2016;13:S422–428. 28005419 10.1513/AnnalsATS.201602-095AW PMC5291468

[30] Akha AA. Aging and the immune system: an overview. J Immunol Methods 2018;463:21–26. 30114401 10.1016/j.jim.2018.08.005

[31] Leone G Voso MT Sica S. Therapy related leukemias: susceptibility, prevention and treatment. Leuk Lymphoma 2001;41:255–76. 11378539 10.3109/10428190109057981

[32] Baltussen JC de Glas NA van Holstein Y. Chemotherapy-related toxic effects and quality of life and physical functioning in older patients. JAMA Network Open 2023;6:e2339116–. 37870832 10.1001/jamanetworkopen.2023.39116 PMC10594146

[33] Le Saux O Falandry C. Toxicity of cancer therapies in older patients. Curr Oncol Rep 2018;20:64. 29896642 10.1007/s11912-018-0705-y

[34] Chang SF Lin HC Cheng CL. The relationship of frailty and hospitalization among older people: evidence from a meta-analysis. J Nurs Scholarsh 2018;50:383–91. 29874399 10.1111/jnu.12397

[35] Abel GA Klepin HD. Frailty and the management of hematologic malignancies. Blood, J Am Soc Hematol 2018;131:515–24. 10.1182/blood-2017-09-746420 29141942

[36] Davis M Fernandez C Vithalani N. Symptoms in advanced hematologic malignancies and other serious hematologic conditions. In: Ullrich CK, Roeland EJ, eds. Palliative Care in Hematologic Malignancies and Serious Blood Disorders: A Clinical Guide. Cham: Springer International Publishing; 2023:169–96. 10.1007/978-3-031-38058-7_14.

[37] Cella D Nowinski CJ Frankfurt O. The impact of symptom burden on patient quality of life in chronic myeloid leukemia. Oncology 2014;87:133–47. 25012261 10.1159/000362816

[38] Shanafelt TD Bowen D Venkat C. Quality of life in chronic lymphocytic leukemia: an international survey of 1482 patients. Br J Haematol 2007;139:255–64. 17897301 10.1111/j.1365-2141.2007.06791.x

[39] Boyd C Smith CD Masoudi FA. Decision making for older adults with multiple chronic conditions: executive summary for the American geriatrics society guiding principles on the care of older adults with multimorbidity. J Am Geriatr Soc 2019;67:665–73. 30663782 10.1111/jgs.15809

[40] Badger T Segrin C Crane T. Social determinants of health, psychological distress, and caregiver burden among informal cancer caregivers of cancer survivors during treatment. J Psychosoc Oncol 2024;42:333–50. 37609806 10.1080/07347332.2023.2248486 PMC10884349

[41] El-Jawahri AR Abel GA Steensma DP. Health care utilization and end-of-life care for older patients with acute myeloid leukemia. Cancer 2015;121:2840–48. 25926135 10.1002/cncr.29430 PMC4418225

[42] Extermann M Aapro M Bernabei R. Use of comprehensive geriatric assessment in older cancer patients:: recommendations from the task force on CGA of the International Society of Geriatric Oncology (SIOG). Crit Rev Oncol Hematol 2005;55:241–52. 16084735 10.1016/j.critrevonc.2005.06.003

[43] Dharmarajan TS. Comprehensive geriatric assessment. In: Springer New York, ed. Geriatric Gastroenterology. Cham: Springer International Publishing; 2021:1–46.

[44] Li CJ Gong SM Shi YJ. Application of comprehensive geriatric assessment in oncology nursing: a literature review on optimizing treatment decisions and patient outcomes. World J Clin Oncol 2025;16:104785. 40290689 10.5306/wjco.v16.i4.104785 PMC12019282

[45] Massaro F Andreozzi F Vandevoorde C. Supportive care in older lymphoma patients to reduce toxicity and preserve quality of life. Cancers (Basel) 2023;15:5381. 38001641 10.3390/cancers15225381 PMC10670135

[46] Vanbutsele G Pardon K Van Belle S. Effect of early and systematic integration of palliative care in patients with advanced cancer: a randomised controlled trial. Lancet Oncol 2018;19:394–404. 29402701 10.1016/S1470-2045(18)30060-3

[47] Seymour J. The impact of public health awareness campaigns on the awareness and quality of palliative care. J Palliat Med 2018;21:S–30. 10.1089/jpm.2017.0391 PMC5733664 29283867

[48] Williams AP Lum JM Deber R. Aging at home: integrating community-based care for older persons. Healthc Pap 2009;10:8–21. 20057212 10.12927/hcpap.2009.21218

[49] Law LY Uong SP Vempaty HT. Regionalization of acute myeloid leukemia treatment in a community-based population: implementation and early results. Perm J 2021;25:20–71. 10.7812/TPP/20.271 PMC8784060 33970088

[50] Wagle KC Skopelja EN Campbell NL. Caregiver-based interventions to optimize medication safety in vulnerable elderly adults: a systematic evidence-based review. J Am Geriatr Soc 2018;66:2128–35. 30136714 10.1111/jgs.15556 PMC6235742

[51] Leider JP Meit M McCullough JM. The state of rural public health: enduring needs in a new decade. Am J Public Health 2020;110:1283–90. 32673103 10.2105/AJPH.2020.305728 PMC7427223

[52] Kivits J Erpelding ML Guillemin F. Social determinants of health-related quality of life. Rev Epidemiol Sante Publique 2013;61:S189–94. 23849946 10.1016/j.respe.2013.06.001

[53] Webb J. Social aspects of chronic transfusions: addressing social determinants of health, health literacy, and quality of life. Hematol Educ Program Am Soc Hematol 2020;2020:175–83. 10.1182/hematology.2020000104 PMC7727521 33275666

[54] Shah E Rodriguez AM Luo JW. Individual-level social determinants of health (SDOH) measures and treatment complications in acute Leukemia. Blood 2023;142:374.

[55] Totadri S Trehan A Kaur A. Effect of socio-economic status & proximity of patient residence to hospital on survival in childhood acute lymphoblastic leukaemia. Indian J Med Res 2019;149:26–33. 31115371 10.4103/ijmr.IJMR_579_17 PMC6507537

[56] Charkhchi P Fazeli Dehkordy S Carlos RC. Housing and food insecurity, care access, and health status among the chronically ill: an analysis of the behavioral risk factor surveillance system. J Gen Intern Med 2018;33:644–50. 29299816 10.1007/s11606-017-4255-z PMC5910337

[57] Arab M Bernstein C Haghshenas A. Factors associated with caregiver burden for mothers of children undergoing Acute Lymphocytic Leukemia (ALL) treatment. Palliat Support Care 2020;18:405–12. 31727187 10.1017/S1478951519000853

[58] Alfano CM Leach CR Smith TG. Equitably improving outcomes for cancer survivors and supporting caregivers: a blueprint for care delivery, research, education, and policy. CA Cancer J Clin 2019;69:35–49. 30376182 10.3322/caac.21548

[59] Levie K Cromartie B Wicks M. Quality assurance and technology-enabled curation of oncology real-world data: the importance of individual quality reviews. J Registry Manag 2023;50:60. 37575551 PMC10414195

[60] Mikhael J Darlington D Howell B. The benefits of telehealth in promoting equity in blood cancer care–results of a multi-stakeholder forum and systematic literature review. J Med Econ 2025;28:788–802. 40340653 10.1080/13696998.2024.2438561

[61] Kobeissi MM Hickey JV. An infrastructure to provide safer, higher-quality, and more equitable telehealth. The Joint Commission Journal on Quality and Patient Safety 2023;49:213–22. 36775714 10.1016/j.jcjq.2023.01.006 PMC9839454

[62] Presley CJ Krok-Schoen JL Wall SA. Implementing a multidisciplinary approach for older adults with cancer: geriatric oncology in practice. BMC Geriatr 2020;20:231. 32631254 10.1186/s12877-020-01625-5 PMC7336473

[63] Board on Global Health, Committee on measuring the impact of interprofessional education on collaborative practice, patient outcomes. measuring the impact of interprofessional education on collaborative practice and patient outcomes.

[64] Dilley JA Bekemeier B Harris JR. Quality improvement interventions in public health systems: a systematic review. Am J Prev Med 2012;42:S58–71. 22502926 10.1016/j.amepre.2012.01.022

[65] Barrientos JC. Management of chronic lymphocytic leukemia in the elderly. Cancer Control 2015;22:17–23. 26618342 10.1177/107327481502204s04 PMC4763599

[66] Hickman LD Phillips JL Newton PJ. Multidisciplinary team interventions to optimise health outcomes for older people in acute care settings: a systematic review. Arch Gerontol Geriatr 2015;61:322–29. 26255065 10.1016/j.archger.2015.06.021

[67] Speer DC Schneider MG. Mental health needs of older adults and primary care: opportunity for interdisciplinary geriatric team practice. Clin Psychol Sci Pract 2003;10:85.

[68] Kurtin S. Interdisciplinary management of acute leukemia across the continuum of care. Semin Oncol Nurs 2019;35:150953. 31748172 10.1016/j.soncn.2019.150953

[69] Goede V Stauder R. Multidisciplinary care in the hematology clinic: implementation of geriatric oncology. J Geriatr Oncol 2019;10:497–503. 30241779 10.1016/j.jgo.2018.09.003

[70] Caserta S Cancemi G Loreta S. Hematological malignancies in older patients: focus on the potential role of a geriatric assessment management. Diagnostics 2024;14:1390. 39001280 10.3390/diagnostics14131390 PMC11241324

[71] Kalsi T Babic-Illman G Ross PJ. The impact of comprehensive geriatric assessment interventions on tolerance to chemotherapy in older people. Br J Cancer 2015;112:1435–44. 25871332 10.1038/bjc.2015.120 PMC4453673

[72] Fernando GV Hughes S. Team approaches in palliative care: a review of the literature. Int J Palliat Nurs 2019;25:444–51. 31585054 10.12968/ijpn.2019.25.9.444

[73] Wang C Zhao B Fang P. Evaluation of the effect of social care collaboration on the psychological state of the parents of children with leukemia. SAGE Open 2024;14:21582440241279568.

[74] Elizondo Rodriguez N Ambrosio L La Rosa-Salas V. Role of the nurse in the design, delivery, monitoring and coordination of cancer survivorship care plans: an integrative review. J Adv Nurs 2022;78:48–62. 34235775 10.1111/jan.14962

[75] Mazanec P Lamb G Haas S. Palliative nursing summit: nurses leading change and transforming care: the nurse’s role in coordination of care and transition management. J Hosp Palliat Nurs 2018;20:15–22. 30063609 10.1097/NJH.0000000000000413

[76] Madu C Mofiyinfoluwa AV Patient-centered care in precision medicine and end-of-life care in neuro-oncology: the role of nursing in enhancing quality of life and treatment outcomes.

[77] Luo X Zhang Y, and Chen Q. Nursing care plan and management of patients with acute leukemia. Altern Ther Health Med 2022;28:80–85. 34653028

[78] Chasen MR Dippenaar AP. Cancer nutrition and rehabilitation—its time has come! Curr Oncol 2008;15:117. 18596892 10.3747/co.v15i3.244 PMC2442766

[79] Silver JK Baima J Newman R. Cancer rehabilitation may improve function in survivors and decrease the economic burden of cancer to individuals and society. Work 2013;46:455–72. 24125901 10.3233/WOR-131755

[80] Strekalova YA Hawkins KE Drusbosky LM. Using social media to assess care coordination goals and plans for leukemia patients and survivors. Transl Behav Med 2018;8:481–91. 29800400 10.1093/tbm/ibx075

[81] Ijiga AC Enyejo LA Odeyemi MO. Integrating community-based partnerships for enhanced health outcomes: a collaborative model with healthcare providers, clinics, and pharmacies across the USA. Open Access Res J Biol Pharm 2024;10:081–104.

[82] Morey T Scott M Saunders S. Transitioning from hospital to palliative care at home: patient and caregiver perceptions of continuity of care. J Pain Symptom Manage 2021;62:233–41. 33385479 10.1016/j.jpainsymman.2020.12.019

[83] Bennardi M Diviani N Gamondi C. Palliative care utilization in oncology and hemato-oncology: a systematic review of cognitive barriers and facilitators from the perspective of healthcare professionals, adult patients, and their families. BMC Palliat Care 2020;19:47. 32284064 10.1186/s12904-020-00556-7 PMC7155286

[84] Flieger SP Chui K Koch-Weser S. Lack of awareness and common misconceptions about palliative care among adults: insights from a national survey. J Gen Intern Med 2020;35:2059–64. 32157652 10.1007/s11606-020-05730-4 PMC7351936

[85] Gerhart J Oswald LB McLouth L. Understanding and addressing mental health disparities and stigma in serious illness and palliative care. Illn Crisis Loss 2025;33:109–29. 10.1177/10541373231201952 PMC11633853 39668846

[86] Bandieri E Borelli E Gilioli F. Stigma of palliative care among patients with advanced cancer and their caregivers on early palliative care. Cancers (Basel) 2023;15:3656. 37509317 10.3390/cancers15143656 PMC10377431

[87] Wedding U. Palliative care of patients with haematological malignancies: strategies to overcome difficulties via integrated care. Lancet Healthy Longev 2021;2:e746–53. 36098031 10.1016/S2666-7568(21)00213-0

[88] Salins N Oncologists’ and Haematologists’ Views of What Facilitates or Hinders Referral of a Child with Advanced Cancer to Palliative Care in India. Lancaster University (United Kingdom); 2021.

[89] Caglayan A Redmond S Rai S. The integration of palliative care with oncology: the path ahead. Ann Palliat Med 2023;12:1373–81. 37872127 10.21037/apm-22-1154

[90] Bowman BA Twohig JS Meier DE. Overcoming barriers to growth in home-based palliative care. J Palliat Med 2019;22:408–12. 30589624 10.1089/jpm.2018.0478

[91] Tomasone JR Brouwers MC Vukmirovic M. Interventions to improve care coordination between primary healthcare and oncology care providers: a systematic review. Esmo Open 2016;1:e000077. 27843639 10.1136/esmoopen-2016-000077 PMC5070279

[92] Crawley LM. Racial, cultural, and ethnic factors influencing end-of-life care. J Palliat Med 2005;8:s–58. 10.1089/jpm.2005.8.s-58 16499470

[93] Nelson KE Wright R Peeler A. Sociodemographic disparities in access to hospice and palliative care: an integrative review. Am J Hosp Palliat Med 2021;38:1378–90. 10.1177/1049909120985419 PMC8514114 33423532

[94] Bala-Hampton JE Dudjak L Albrecht T. Perceived economic hardship and distress in acute myelogenous leukemia. J Oncol Navig Surviv 2017;8:258–64.

[95] Stubbins RJ Stamenkovic M Roy C. Incidence and socioeconomic factors in older adults with acute myeloid leukaemia: real-world outcomes from a population-based cohort. Eur J Haematol 2022;108:437–45. 35122325 10.1111/ejh.13752

[96] Hughes MC Vernon E Hainstock A. The effectiveness of community-based palliative care programme components: a systematic review. Age Ageing 2023;52:afad175. 37740895 10.1093/ageing/afad175 PMC10517647

[97] Kamal AH Currow DC Ritchie CS. Community-based palliative care: the natural evolution for palliative care delivery in the US. J Pain Symptom Manage 2013;46:254–64. 23159685 10.1016/j.jpainsymman.2012.07.018

[98] Agha RA Mathew G Rashid R, TITAN Group. Transparency in the reporting of Artificial Intelligence – the TITAN guideline. Premier J Sci 2025;10:100082.

